# P-440. Shortened steroid regimen in people living with HIV/AIDS with associated moderate and severe *P. jirovecii* pneumonia

**DOI:** 10.1093/ofid/ofae631.640

**Published:** 2025-01-29

**Authors:** Amy Bethel Peralta Prado, Xavier A Flores-Andrade, María I León-Rodríguez, Víctor H Ahumada-Topete, Santiago Ávila-Ríos

**Affiliations:** Instituto Nacional de Enfermedades Respiratorias, MEXICO, Distrito Federal, Mexico; Instituto Nacional de Enfermedades Respiratorias, MEXICO, Distrito Federal, Mexico; Instituto Nacional de Enfermedades Respiratorias, MEXICO, Distrito Federal, Mexico; Instituto Nacional de Enfermedades Respiratorias, MEXICO, Distrito Federal, Mexico; National Institute of Respiratory Diseases, Mexico City, Distrito Federal, Mexico

## Abstract

**Background:**

The 21-day steroid regimen in people with *P. jirovecii* pneumonia (PCP) showed positive clinical outcomes reducing mortality and immune reconstitution inflammatory syndrome (IRIS) incidence. However, steroids can perpetuate an immunocompromised state, having a negative impact on morbidity.

Respiratory Function test in PLWHIV with shortened vs conventional steroid regimen for PCP

**Figure:**
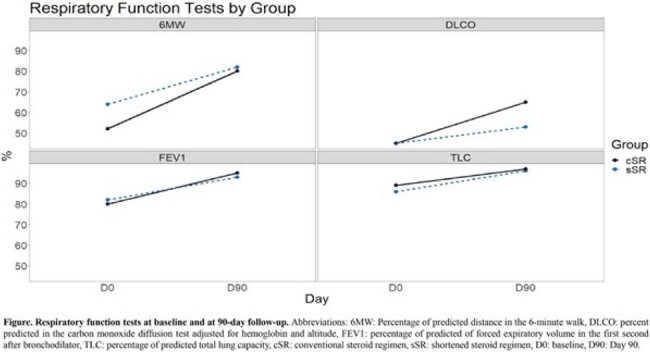

**Methods:**

This is an open-label, randomized non-inferiority controlled trial in adults living with HIV, to elucidate the effect of a shortened steroid regimen (sSR) of 8 days for moderate and 14 days for severe PCP compared to conventional 21-day steroid regimen (cSR) by mortality, IRIS incidence, and respiratory function. Herpesviruses and HIV viral loads, CD4 T cells (CD4), and respiratory function tests were performed at admission and at day 90.

**Results:**

A total of 44 participants were randomized, 23 in sSR and 21 in cSR. Mechanical ventilation (MV) was equal between groups (13 participants each group (OR = 0.8, 95%CI = 0.24-2.67). HIV viral loads at admission and at day 90 were similar between groups (*p* = 0.34 and p = 0.33 respectively). Similarly, CD4 did not show differences at admission and day 90 (*p* = 0.82 and *p* = 0.73). Respiratory function tests were similar between both groups (Figure attached). The CMV, EBV, and HHV-8 viral loads did not show statistical differences. A total of 3 participants presented IRIS in sSR vs 2 in cSR (OR = 1.5, 95%CI = 0.23-9.96) and mortality was not statistically different (OR = 3, 95%CI = 0.29-31.35). The sSR group had a shorter hospital stay (12 vs 18 days; *p* = 0.37).

**Conclusion:**

Preliminary analysis shows that reducing steroid duration may be safe, since a sSR achieved non-inferiority compared with a cSR, by presenting similarities in mortality, IRIS incidence, hospital-stay duration, MV requirements, herpesviruses and HIV viral loads, and in respiratory function tests; suggesting that at 90 days follow-up, the use of a sSR is not inferior compared to cSR in this preliminary analysis.

**Disclosures:**

**All Authors**: No reported disclosures

